# Digital Information Technology Use and Patient Preferences for Internet-Based Health Education Modalities: Cross-Sectional Survey Study of Middle-Aged and Older Adults With Chronic Health Conditions

**DOI:** 10.2196/12243

**Published:** 2019-04-04

**Authors:** Nancy P Gordon, Elizabeth Crouch

**Affiliations:** 1 Division of Research Kaiser Permanente Northern California Oakland, CA United States; 2 Department of Health Services Policy & Management University of South Carolina Columbia, SC United States

**Keywords:** internet, health status disparities, aged, health informatics, information technology, health education

## Abstract

**Background:**

Health information, patient education, and self-management (health information and advice, HIA) tools are increasingly being made available to adults with chronic health conditions through internet-based health and mobile health (mHealth) digital information technologies. However, there is limited information about patient preferences for using specific types of health information and advice resources and how preferences and usage differ by age group and education.

**Objective:**

The objective of this study was to examine how use of digital information technologies and preferred methods for obtaining health information and advice varies by age group and education among middle-aged and older adults with chronic health conditions.

**Methods:**

The study used cross-sectional survey data for 9005 Kaiser Permanente Northern California members aged 45 to 85 years who responded to a mailed and Web-based health survey conducted during 2014 and 2015 and indicated having at least 1 chronic health condition. Bivariate analyses and logistic regression models with weighted data were used to estimate and compare the prevalence of digital information technology use, past-year use of internet-based health information and advice resources, and preferences for using internet-based, mHealth, and traditional health information and advice modalities for adults aged 45 to 65 years, 66 to 75 years, and 76 to 85 years.

**Results:**

The percentages of adults who used digital information technologies (computers, smartphones, internet, email, and apps), had obtained health information and advice from an internet-based resource in the past year, and who were interested in using internet-based and mHealth modalities for obtaining health information and advice declined with age. Within age group, prevalence of digital information technologies use and interest in internet-based and mHealth modalities was lower among adults with no college education versus college graduates. Differences in preferences for internet-based health information and advice modalities between the oldest and younger groups and those with lower versus higher education were substantially diminished when we restricted analyses to internet users.

**Conclusions:**

Health care providers and organizations serving middle-aged and older adults with chronic health conditions should not assume that patients, especially those who are older and less educated, want to engage with internet-based and mHealth resources. In addition, increasing the engagement of nonutilizers of digital devices and the internet with internet-based health information and advice and mHealth apps might require both instrumental (eg, providing digital information technology devices, internet, and skills training) and social support. As part of patient-centered care, it is important for providers to ascertain their patients’ use of digital information technologies and preferences for obtaining health information and patient education rather than routinely referring them to internet-based resources. It is also important for health care providers and consumer health organizations to user test their Web-based resources to make sure they are easy for older and less educated adults to use and to make sure that it remains easy for adults with chronic conditions to obtain health information and patient education using offline resources.

## Introduction

The prevalence of internet use among US seniors (ages ≥65 years) has been increasing, partly because of the aging of *Baby Boomers* into the older adult group [1]. However, surveys of US adults have consistently found that seniors are significantly less likely than middle-aged adults to be using the internet [2-4]. Data from the 2017 US Current Population Survey Computer and Internet Use Supplement (CPS-CIUS) suggest that in 2017, approximately 79% of middle-aged adults and 62% of seniors were using the internet, up from 77% and 56%, respectively, in 2015 [5,6]. Although the overall rate of senior usage was higher, there was a large disparity in internet use between younger seniors (aged 65 to 74 years, 70%) and older seniors (52% of those aged 75 to 84 years and 38% of those aged ≥85 years). Other surveys have confirmed this finding of lower prevalence of internet use among older versus younger seniors [2,4,6-11], those with higher levels of educational attainment versus those with lower levels of educational attainment [4,7-14], and among blacks and Hispanics as compared with non-Hispanic whites [4,7-10,14-17]. There is also some evidence that most seniors who are not already using the internet are unlikely to start doing so in the future [2].

Many middle-aged and older adults are using the internet to obtain information about health conditions and treatments, to get social support and advice from others with similar health-related experiences and to access apps to help them manage their health [15,18-21]. The CPS-CIUS 2015 survey showed that slightly over half of the middle-aged and older adult internet users in the United States had searched for health information online in the past year, which translates into approximately 39% of all middle-aged adults, 31% of all younger seniors (aged 65 to 74 years), and 23% of all older seniors (aged 75 to 84 years) [5]. These latter estimates are only slightly higher than Choi’s estimates of 32% of adults aged 65 to 74 years and 14% of adults aged 75 to 84 years, on the basis of the 2009 National Health Interview Survey [7]. In addition to age, studies have shown racial/ethnic and educational disparities in the use of the internet to obtain health information [3,8,22,23].

There has been a burgeoning of health information websites, online interactive health programs, health-related forums, podcasts, and health apps on the internet since the early 2000s [18,19,24]. For many reasons, including marketing [25], consumer demand [26], federal regulations and incentive programs [27], and a growing body of evidence about improvement in patient engagement and health outcomes [28,29], health care providers and health organizations have begun to use the internet as a primary platform for providing information and advice on health and medical topics [30]. Approximately 60% of US adults have at least one chronic health condition, and this percentage is expected to increase as the population ages [31]. Internet users with chronic health conditions are more likely than other internet users to access health information online [32,33], and internet-based resources will become increasingly important tools for chronic conditions management (CCM) [34-38]. However, this shift to greater reliance on digital platforms for patient education and monitoring and patient-provider communication will potentially make it more difficult for older and less educated adults with chronic health conditions to obtain health information as they are less likely to have the digital technology (eg, Web-enabled devices, high speed internet) and skills and confidence to use the internet [4,37]. There is also some evidence that suggests many adults who use the internet might still prefer to obtain health information and advice (HIA) using more traditional methods, including print materials and oral communication with health care professionals [8,23,32,39-41].

Although there has been extensive research on patient portal use by middle-aged and older adults [38,42-48], there is less information about the use of internet-based health information and patient education resources by these age groups [7,23,49-52]. Given the trend of health information and patient education programs migrating to websites and other digital platforms, it is important for health care providers and organizations to have an awareness of digital information technology (DIT) use and preferred modalities for obtaining HIA among middle-aged and older adults with chronic health conditions as patient-centered care services for chronic conditions, including CCM programs and patient-facing health education resources, are being developed and implemented. In an earlier paper, we described the prevalence and factors influencing the use of the internet, patient portal, and online health information resources by middle-aged and older adult members of a large Northern California health plan [53]. In this paper, we describe DIT use and HIA modality preferences of middle-aged and older adult members of the same health plan who reported having at least one chronic health condition. We show how in this insured population, the use of DIT and interest in using internet-based HIA resources and apps differs by age group (45 to 65 years, 66 to 75 years, and 76 to 85 years), and within age groups, by level of education.

## Methods

### Setting

The Kaiser Permanente Medical Care Program in Northern California (KPNC) provides primary and specialty health care to a sociodemographically diverse membership that includes over 2.8 million adults who mostly reside in the San Francisco Bay Area, Sacramento area, Silicon Valley, and Central Valley. The KPNC adult membership is very similar to the insured population of Northern California with regard to sociodemographic and health characteristics [54]. For several years, the health plan has had a comprehensive website accessible to members and the general public, which provides information and advice about health conditions, medical procedures, medications and dietary supplements, and health and lifestyle risks and behavior change using online text, video, and podcasts, as well as online health behavior change programs available to members who register to use the patient portal.

### Survey Sample

Data for this study come from middle-aged and older adults who participated in the 2014 to 2015 cycle of the KPNC Member Health Survey (MHS). The MHS is a self-administered (mailed print questionnaire and online) survey that has been conducted with independent stratified random samples of English-speaking adults every 3 years since 1993. The survey covers sociodemographic and health-related characteristics, digital technology use, use of the patient portal and different types of health information resources during the previous 12 months, and preferred methods for obtaining information and advice about managing health conditions and making changes in health-related behaviors and lifestyle. Information about the survey is found in an earlier publication [55] and on the survey website [56]. The overall response rate for this age group in the 2014 to 2015 survey cycle was 49.3% (40.9% for those aged 45 to 65 years and 64.5% for those aged 66 to 85 years).

The sample used for these analyses was restricted to the 9005 respondents (4163 aged 45 to 65 years, 2656 aged 66 to 75 years, and 2186 aged 76 to 85 years) who were not missing data on internet use status and who indicated having at least 1 of the following chronic health conditions during the previous year: diabetes, prediabetes, high blood pressure, heart condition, high cholesterol, cancer, Parkinson’s disease, urinary incontinence, chronic obstructive pulmonary disease/chronic bronchitis, asthma, allergies, musculoskeletal pain, osteoarthritis, frequent migraines or other types of headache, chronic pain, frequent insomnia, depression, anxiety, frequent memory problems, or frequent problems with balance or walking. This subsample includes 81.1% of all respondents aged 45-85 years, with 93% of exclusions because of not meeting the chronic health condition criterion.

### Study Variables

#### Sociodemographic Characteristics

These included age group (45 to 65 years, 66 to 75 years, and 76 to 85 years for age group comparisons), sex (female, male), race/ethnicity (white, black, Latino, Filipino, East Asian, other Asian, Pacific Islander, other), educational attainment (no college, some college or community college degree, bachelor’s or postgraduate degree), and household income (HHI in US ≤$25,000, $25,001-$35,000, $35,001-$50,000, $50,001-$65,000, $65,001-$80,000, $80,001-$100,000, >$100,000).

#### Digital Information Technology Access and Use

The digital information technologies studied included having a mobile phone, smartphone, easy access to a computer or tablet, uses the internet (with, without help from another person) to get information from websites, uses email (with, without help from another person), able to send and receive text messages, able to use apps on a smartphone.

#### Use of Internet-Based and Noninternet-Based Health Information and Advice Resources in the Past 12 Months

Web-based HIA users were those individuals who reported obtaining HIA from the kp.org or another website, using a kp.org Web-based patient education program (eg, preparing for a procedure, health calculator, or health lifestyle programs for nutrition, weight, stress, or exercise) or podcast, or participating in any online chat room or community related to a health condition. Noninternet-based HIA users were those who indicated participating in any KPNC group or individual health education program/service or used KPNC print health education materials. Individuals who reported using any of the 2 categories of HIA resources were considered to have used any HIA resource.

#### Interest in Using Internet-Based Health Information and Advice Modalities, Health Apps, and Noninternet-Based Health Information and Advice Modalities

Individuals were asked to indicate whether they would like to get information and advice about how to manage health conditions and to make changes in health behaviors (diet, exercise, etc) using 1 or more internet-based and more traditional health education modalities. The checklist included 9 internet-based modalities (getting information from websites and/or doctor’s home page on the kp.org website, watching Web-based videos, watching live webinars or Web-based talks, listening to a podcast or online audio program, using a Web-based interactive program, emailed newsletters, getting HIA through a secure patient portal message, having a video visit with a patient educator, or joining an online chat room or online support community), health apps, and 7 more traditional/noninternet-based HIA modalities (telephone and in-person counseling sessions with a patient educator, in-person workshops and multi-session classes, DVDs, interactive computer programs, print materials, mailed health newsletters, and text messages).

### Data Analysis

All analyses were performed using SAS version 9.4 (SAS Institute, Cary, NC 2013) procedures for data from complex survey designs [57] and data weighted to the age, sex, and geographic composition of the KPNC adult membership in 2014. Proc Surveyfreq was used to produce weighted percentages with 95% confidence levels and Proc Surveylogistic was used to test whether differences between age groups and levels of educational attainment in access to digital devices and use of and interest in different HIA modalities were statistically significant after adjusting for race/ethnicity and sex. Analyses of patient-preferred HIA modalities were restricted to individuals who indicated interest in at least one modality in the HIA checklist. All differences between subgroups mentioned in the text are statistically significant at *P*<.05 or greater; if differences are not mentioned, they did not reach that threshold. Although we did not adjust for multiple comparisons, we have reported results of all statistical comparisons.

### Ethics

Use of MHS data for this study was approved by KPNC’s Institutional Review Board.

## Results

### Characteristics of Survey Respondents

The sociodemographic characteristics of the middle-aged (45 to 65 years), younger senior (66 to 75 years), and older senior (76 to 85 years) study groups are shown in Table 1. Slightly over half of all 3 age groups are female. Compared with middle-aged adults, the 2 senior groups are significantly (*P*<.001) more likely to be non-Hispanic white, and by San Francisco Bay Area standards, more likely (*P*<.001) to be lower income (HHI≤US $35,000) and less likely to have an HHI>US $80,000, with older seniors being less financially well-off than the younger seniors. Older seniors are significantly (*P*<.001) more likely than the middle-aged and younger senior groups to have no college (a high school diploma or less education) and less likely to be college graduates (bachelor’s or postgraduate degree).

### Digital Information Technology Use

Table 2 provides data on access to digital technology by age group and level of education. Compared with middle-aged adults, after controlling for sex and race/ethnicity, both senior groups are significantly (*P*<.001) less likely to have easy access to a computer (desktop or laptop), to own a smartphone, and to be using the internet, email, text messaging, and health apps, and older seniors are significantly (*P*<.001) less likely than younger seniors to be engaging with these digital technologies.

**Table 1:** Study sample characteristics.

Characteristics			45 to 65 years, n (%)^a^	66 to 75 years, n (%)	76 to 85 years, n (%)
**Sex**					
	Male		1905 (46.9)	1286 (45.7)	1161 (45)
	Female		2258 (53.1)	1370 (54.3)	1025 (55)
**Race/ethnicity**					
	White non-Hispanic		2419 (58.6)	1867 (71.9)^b^	1541 (73.4)^b^
	Black		359 (7.7)	175 (6.1)	141 (6.4)
	Hispanic		584 (13.8)	206 (6.9)	219 (8.8)
	Filipino		250 (5.9)	148 (5.2)	99 (3.9)
	East Asian		349 (8.4)	148 (5.4)	121 (4.9)
	Other Asian		91 (2.8)	49 (2.2)	24 (0.7)
	Other		111 (2.7)	63 (2.2)	41 (1.9)
**Education**					
	**No college**		921 (22.2)	633 (21.5)	765 (38.4)^b,c^
		< High school graduate	112 (2.5)	106 (3.2)	174 (9.1)
		High school graduate	809 (19.7)	527 (18.3)	591 (29.3)
	Some college/AA degree		1481 (35.2)	910 (35.3)	675 (29.5)
	College graduate (Bachelor’s degree or higher)		1739 (42.6)	1087 (43.2)	724 (32.1)^b,c^
**Household income (US$)**					
	≤$25,000		336 (8.1)	325 (12.0)^b^	428 (24.5)^b,c^
	$25,000-$35,000		216 (4.9)	269 (10.7)	275 (14.4)
	$35,001-$50,000		434 (10.2)	368 (14.6)	368 (19.8)
	$50,001-$65,000		406 (9.9)	281 (11.6)	263 (12.5)
	$65,001-$80,000		482 (12.2)	323 (13.6)	216 (10.2)
	$80,0001-$100,000		601 (14.9)	312 (13.9)	172 (8.5)
	>$100,000		1499 (39.9)	548 (23.6)^b^	225 (10.1)^b,c^

^a^n: unweighted count; %: percentage of age group with this characteristic based on weighted survey data.

^b^Significantly (*P<*.001) different from ages 45 to 65 years.

^c^Significantly (*P<*.001) different from ages 66 to 75 years.

**Table 2:** Use of digital information technologies by age group and level of education.

Digital technology use			45 to 65 years (n=3671)		66 to 75 years (n=2196)			76 to 85 years (n=1707)		
			%^a^	95% CI		%	95% CI		%	95% CI
**All**										
	**Uses the internet to obtain information**									
		Uses by self or with help	95.4	94.8-96.1		88.3^b^	87.0-89.6		67.5^b^	64.7-70.2
		Uses by self	91.3	90.4-92.2		79.9^b^	78.2-81.5		53.6^b,c^	50.8-56.4
	**Uses email**									
		Uses by self or with help	94.9	94.2-95.6		87.7^b^	86.3-89.0		68.9^b,c^	66.2-71.6
		Uses by self	91.5	90.6-92.4		81.2^b^	79.6-82.8		56.1^b,c^	53.3-59.0
	Has access to a computer or laptop		95.8	95.2-96.5		91.3^b^	90.1-92.4		75.7^b,c^	73.2-78.3
	Has a mobile phone		95.9	95.3-96.5		92.3^b^	91.2-93.4		81.1^b,c^	78.8-83.4
	Has a smartphone		70.3	68.8-71.8		45.0^b^	42.9-47.1		18.2^b,c^	16.1-20.3
	**Able to send/receive text messages**		76.1	74.7-77.6		52.8^b^	50.6-54.9		31.8^b,c^	29.1-34.4
		If has a mobile phone	79.4	78.0-80.8		57.2^b^	55.0-59.4		39.2^b,c^	36.1-42.2
	**Able to use apps on a smartphone**		54.5	52.8-56.1		28.3^b^	26.3-30.2		9.8^b,c^	8.2-11.3
		If has a smartphone	77.5	75.9-79.2		62.8^b^	59.7-66.0		53.8^b,d^	47.4-60.1
**No college (high school or less)**										
	**Uses the internet to obtain information**									
		Uses by self or with help	88.0^e^	85.9-90.2		70.3^b,e^	66.4-74.3		45.9^b,c,e^	41.0-50.7
		Uses by self	77.2^e^	74.3-80.2		55.4^b,e^	51.0-59.8		31.1^b,c,e^	26.6-35.6
	**Uses email**									
		Uses by self or with help	87.2^e^	85.0-89.5		69.2^b,e^	65.1-73.2		49.4^b,c,e^	44.5-54.4
		Uses by self	78.1^e^	75.2-81.0		57.7^b^	53.3-62.0		32.7^b,c,e^	28.2-37.2
	Has access to a computer or laptop		88.5^e^	86.2-90.7		77.3^b,e^	73.7-80.9		57.5^b,c,e^	52.6-62.4
	**Has a mobile phone**		95.0^f^	93.5-96.4		90.1^b,g^	87.7-92.6		73.9^b,c,f^	69.4-78.4
		Has a smartphone	56.9^e^	53.4-60.5		25.1^b,e^	21.1-29.0		11.1^b,c,e^	7.9-14.4
	**Able to send/receive text messages**		67.9^e^	64.5-71.3		44.0^b,e^	39.5-48.4		26.6^b,c,g^	22.4-30.9
		If has a mobile phone	71.5^e^	68.1-74.9		48.8^b,e^	44.1-53.5		36.1^b,c^	30.7-41.4
	**Able to use apps on a smartphone**		41.4^e^	37.9-45.0		14.7^b,e^	11.4-17.9		5.0^b,c,e^	2.7-7.3
		If has a smartphone	72.8^g^	68.5-77.1		58.6^h^	49.6-67.5		45.1^b,e^	29.6-60.6
**Some college/AA degree**										
	**Uses the internet to obtain information**									
		Uses by self or with help	95.8^e^	94.8-96.9		89.4^b,e^	87.2-91.6		75.4^b,c,e^	71.1-79.8
		Uses by self	91.8^e^	90.3-93.2		80.9^b,e^	78.1-83.8		61.3^b,c,e^	56.5-66.1
	**Uses email**									
		Uses by self or with help	95.0^e^	93.8-96.1		88.3^b,e^	86.0-90.6		74.8^b,c,e^	70.3-79.3
		Uses by self	92.2^e^	90.8-93.6		82.5^b,e^	79.8-85.2		64.6^b,c,e^	59.8-69.4
	Has access to a computer or laptop		96.0^e^	94.9-97.0		91.6^b,e^	89.6-93.7		82.3^b,c,e^	78.4-86.2
	Has a mobile phone		95.7	94.6-96.8		91.9^i^	90.0-93.9		85.5^b,c^	82.1-89.0
	Has a smartphone		70.3^e^	67.8-72.8		44.5^b,e^	40.8-48.2		20.1^b,c^	16.3-23.9
	**Able to send/receive text messages**		75.2^e^	72.8-77.6		53.0^b,h^	49.4-56.7		34.9^b,c^	30.2-39.6
		If has a mobile phone	78.6^e^	76.2-80.9		57.7^b^	53.9-61.4		40.8^b,c^	35.5-46.1
	**Able to use apps on a smartphone**		53.6^e^	50.8-56.4		27.8^b,e^	24.5-31.2		10.3^b,c,i^	7.5-13.1
		If has a smartphone	76.2^f^	73.4-79.1		62.5^b^	57.1-67.9		51.3^b^	40.8-61.8
**College graduate (Bachelor’s degree or higher)**										
	**Uses the internet to obtain information**									
		Uses by self or with help	99.1	98.7-99.6		96.3^b^	95.2-97.5		86.7^b,c^	83.3-90.1
		Uses by self	98.4	97.8-99.0		91.4^b^	89.6-93.2		73.9^b,c^	69.6-78.3
	**Uses email**									
		Uses by self or with help	98.8	98.3-99.4		96.4^b^	95.2-97.5		86.8^b,c^	83.5-90.2
		Uses by self	97.9	97.2-98.6		91.9^b^	90.2-93.7		76.1^b,c^	71.9-80.3
	Has access to a computer or laptop		99.5	99.2-99.8		97.9^b^	97.1-98.7		91.3^b,c^	88.5-94.1
	Has a mobile phone		96.7	95.8-97.6		93.6^b^	92.1-95.2		85.6^b,c^	82.3-88.8
	Has a smartphone		77.3	75.2-79.4		55.6^b^	52.3-58.8		25.0^b,c^	21.0-29.0
	**Able to send/receive text messages**		81.2	79.3-83.2		57.0^b^	53.7-60.2		35.5^b,c^	30.9-40.2
		If has a mobile phone	84	82.1-85.8		60.8^b^	57.5-64.2		41.5^b,c^	36.3-46.7
	**Able to use apps on a smartphone**		62.1	59.6-64.5		35.3^b^	32.2-38.5		15.0^b,c^	11.9-18.1
		If has a smartphone	80.3	78.0-82.5		63.6^b^	59.3-67.9		60.0^b^	50.9-69.0

^a^N: unweighted count; %: percentage of age group with this characteristic based on weighted survey data.

^b^Significantly (*P<*.001) lower than ages 45 to 65 years after controlling for sex and race/ethnicity.

^c^Significantly (*P<*.001) lower than ages 66 to 75 years after controlling for sex and race/ethnicity.

^d^Significantly (*P<*.01) lower than ages 66 to 75 years after controlling for sex and race/ethnicity.

^e^Significantly (*P<*.001) lower than college graduates in this age group after controlling for sex and race/ethnicity.

^f^Significantly (*P<*.05) lower than college graduates in this age group after controlling for sex and race/ethnicity.

^g^Significantly (*P<*.01) lower than college graduates in this age group after controlling for sex and race/ethnicity.

^h^Significantly (*P<*.01) lower than ages 45 to 65 years after controlling for sex and race/ethnicity.

^i^Significantly (*P<*.05) lower than college graduates in this age group after controlling for sex and race/ethnicity.

Table 2 also shows that across all age groups, noncollege graduates are significantly (*P*<.001) less likely than college graduates to be using these technologies. In addition, the same age group differences are seen at each level of education, with the exception of being able to use apps on a smartphone, which was extremely low prevalence across all groups.

Approximately 62.5% (95% CI 61.2%-63.8%) of adults aged 45 to 75 years and 53.5% (95% CI 50.7%-56.4%) of adults aged 76 to 85 years had in the past 12 months used at least one of the HIA resources asked about in the survey. After controlling for age group, sex, and race/ethnicity, past-year HIA users with no college education were significantly (*P*<.001) less likely than college graduates to have used an internet-based HIA resource—odds ratio (OR) 0.60 (95% CI 0.48-0.74)—whereas those with some college did not significantly differ from college graduates.

### Use of Internet-Based HIA Resources in Past Year

**Figure 1:**
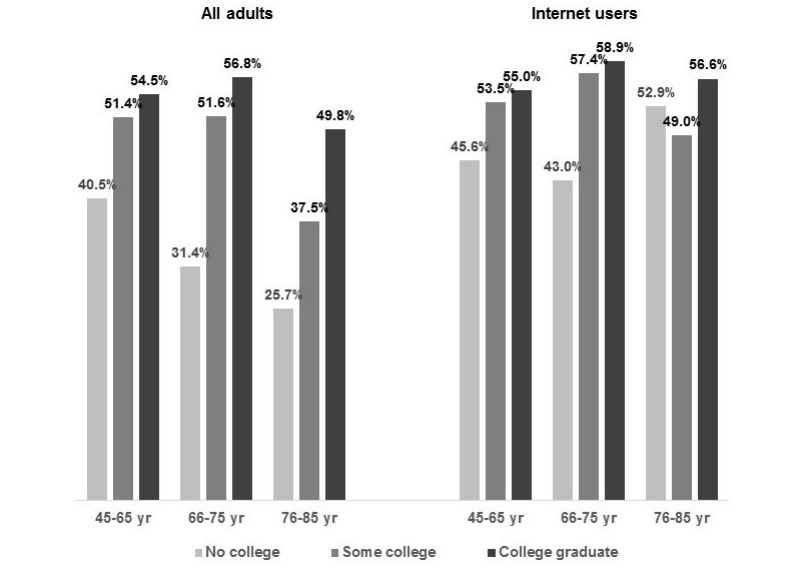
Percentages of middle-aged and older adults who obtained health information from a website in the past 12 months, by level of education, all adults and internet users.

Table 3 provides statistics on use of specific kinds of internet-based HIA resources in the past year by adults in these 3 age groups. Half of the adults aged 45 to 75 years and approximately one-third of those aged 76 to 85 years had obtained HIA from a website. However, no significant age group difference in accessing HIA from websites was observed among internet users. Approximately 10% of adults aged 45 to 75 years and 5% of adults aged 76 to 85 years had used a Web-based health education program, and the age group difference, although smaller, remained statistically significant among internet users. Health app use significantly declined with age (approximately 11% of those aged 45 to 65 years, 5% of those aged 66 to 75 years, and 3% of those aged 76 to 85 years, respectively), with prevalence of use among smartphone users approximately 3% points higher in each age group. Across all age groups, less than 3% of adults had listened to a podcast on the health plan’s website and less than 1% had participated in an online chat room on any website. Figure 1 shows that across all 3 age groups, adults with no college education were significantly (*P*<.001) less likely than college graduates to have obtained HIA from a website, and in the 2 older groups, those with some college were also significantly less likely than college graduates to have obtained Web-based HIA. However, among internet users, education-related differences in obtaining internet-based HIA were greatly diminished in all age groups (see Multimedia Appendix 1 for results of age group-specific multivariable logistic regression models of past year use of HIA from a website). Use of health plan Web-based health education programs and podcasts did not significantly differ by level of education, but among middle-aged and older adults, those with no college education were significantly (*P*<.01) less likely than college graduates to have used health apps (ages 45 to 65 years: 7.8%, 95% CI 5.7%-5.9% vs 12.4%, 95% CI 10.6%-14.2%; ages 66 to 75 years: 2.4%, 95% CI 1.1%-3.6% vs 6.3%, 95% CI 4.7%-8.0%).

### Interest in Using Internet- and Noninternet-Based HIA Modalities

Table 4 shows the percentages of all adults and internet users who indicated interest in obtaining HIA using specific internet- and noninternet-based modalities, restricted to the 86% of people who expressed interest in using at least one HIA modality asked about in the survey. Although 75% of all middle-aged and older adults expressed interest in using at least one of the internet-based HIA modalities, there were significant differences in interest among middle-aged, younger, and older seniors that persisted when restricted to internet users. Among all adults, after controlling for sex and race/ethnicity, 66 to 75 year olds and 76 to 85 year olds were less likely than 45 to 65 year olds to be interested in watching Web-based videos and webinars, using an interactive Web-based program, listening to podcasts, having a video visit with a patient educator, receiving HIA text messages, receiving emailed health newsletters, and using health apps. Adults aged 76 to 85 years were less likely than 45 to 65 year olds to be interested in obtaining HIA in messages sent through the patient portal, reading about health topics on a website, and receiving emailed health newsletters, but 66 to 75 year olds did not differ significantly from the middle-aged group. No age group difference was observed for print materials or counseling over the phone, but 76 to 85 year olds were less interested in counseling or classes that involved coming into the medical facility.

**Table 3:** Use of selected internet-based health information and mobile health resources in the past year, by age group and education.

Modality used in past year		45 to 65 years				66 to 75 years				76 to 85 years			
		%^a^		95% CI		%		95% CI		%		95% CI	
**Any internet-based health resource^b^**		51.9		50.3-53.6		51.1		49.0-53.2		38.1^c,d^		35.3-40.8	
	Internet users^e^		54.3		52.5-56.0		57.4		55.2-59.7		54.8		51.6-58.1
**Information from a website**		50.2		48.5-51.9		49.4		47.3-51.5		36.7^c,d^		34.0-39.5	
	Internet users^e^		52.4		50.7-54.1		55.5		53.2-57.7		53		49.7-56.3
**Web-based health education program**		10.4		9.4-11.5		9.7		8.4-11.0		4.9^c,d^		3.7-6.0	
	Internet users^e^		10.9		9.8-12.0		10.9		9.4-12.3		7.0^c,f^		5.4-8.6
Podcast from health plan website		2.2		1.7-2.7		1.8		1.1-2.4		1.0^g^		0.5-1.5	
**Any health app**		10.7		9.6-11.8		5.2^c^		4.2-6.1		2.7^c,f^		1.8-3.7	
	Smartphone users		13.9		12.4-15.3		8.5^c^		6.7-10.3		7.3^h^		3.1-11.6
Health chat room/online community		0.9		0.6-1.2		0.5		0.2-0.8		0.5		0.1-0.9	

^a^%: percentage of age group with this characteristic based on weighted survey data.

^b^Internet-based health resources included information from a website, online health education program, podcast, or health chat room/online community.

^c^Significantly (*P<*.001) lower than ages 45 to 65 years after controlling for sex and race/ethnicity.

^d^Significantly (*P<*.001) lower than ages 66 to 75 years after controlling for sex and race/ethnicity.

^e^Internet users are those who used the internet on their own or with help. Ns for internet users: ages 45 to 65 years: 1259; ages 66 to 75 years: 699; ages 76 to 85 years: 429.

^f^Significantly (*P<*.01) lower than ages 66 to 75 years after controlling for sex and race/ethnicity.

^g^Significantly (*P<*.01) lower than ages 45 to 65 years after controlling for sex and race/ethnicity.

^h^Significantly (*P*<.05) lower than ages 45 to 65 years after controlling for sex and race/ethnicity.

**Figure 2:**
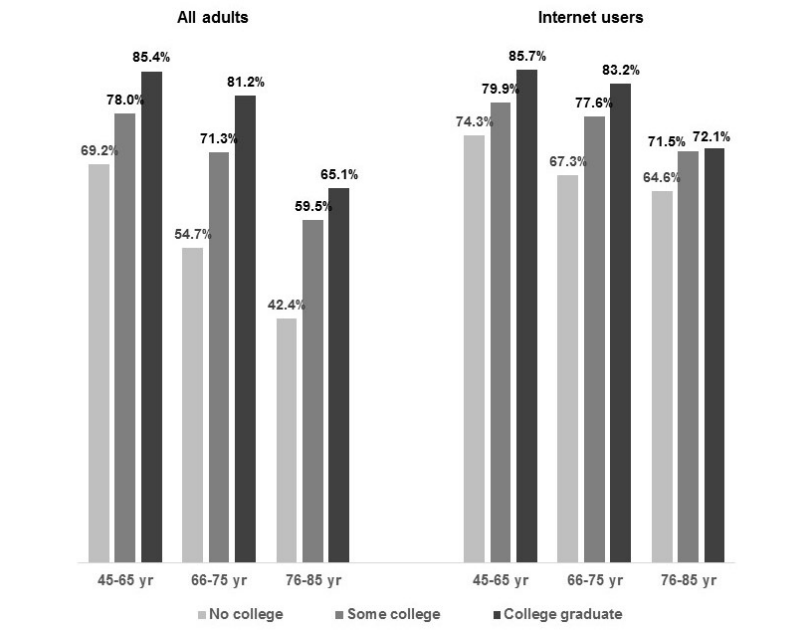
Percentages of middle-aged and older adults interested in using Web-based resources to obtain health information and advice, by level of education, all adults and internet users.

Figure 2 shows that across all age groups, adults with no college education were significantly (*P*<.001) less likely than college graduates to be interested in using at least one internet-based HIA modality, and among middle-aged and younger seniors, those with some college were also significantly less likely than college graduates to be interested in using any internet-based HIA modality. In Multimedia Appendix 2, we show that at all age levels, adults with no college education are significantly less likely than college graduates to prefer online HIA modalities.

As 97% of 45 to 65 year olds and 91% of 66 to 75 year olds interested in at least one HIA modality were internet users, prevalence of interest in using internet-based HIA modalities did not significantly differ between internet users and nonusers in those age groups. However, as only 73% of 76 to 85 year olds were internet users, interest in obtaining HIA from websites, patient portal messages, and emailed newsletters was significantly higher among online adults. Across all age groups, prevalence of interest in using health apps was significantly higher among smartphone users than all adults, but prevalence of interest in HIA text messages was not significantly higher among adults who currently use text messaging than all adults.

**Figure 3:**
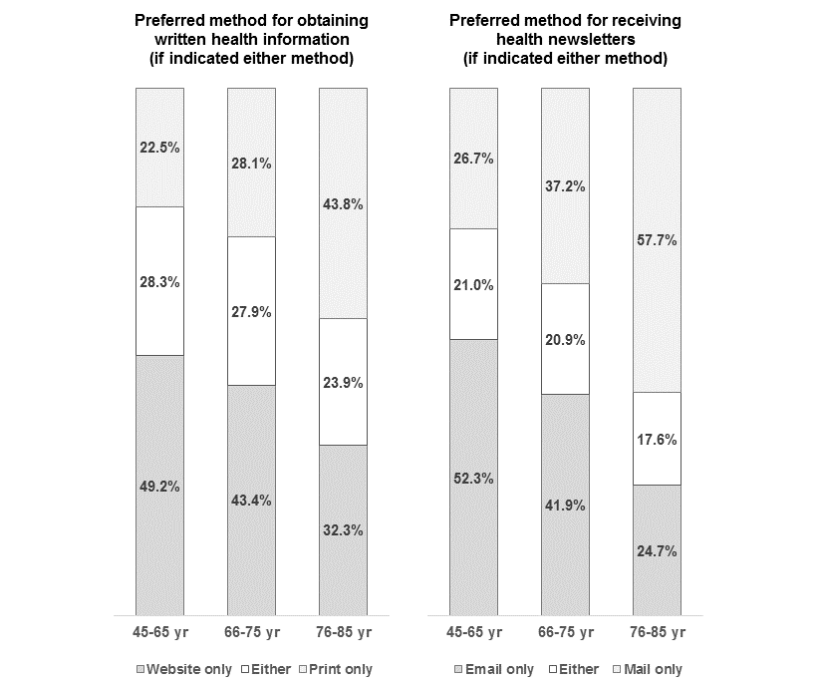
Differences by age group in preferred methods for receiving written health information and health newsletters.

Figure 3 shows how the preference for obtaining textual HIA and health newsletters varies by age among those interested in textual HIA and health newsletters. Among those interested in textual information, preference for obtaining it only from print materials significantly increased with age (ages 66 to 75 vs 45 to 65: OR 1.44, 95% CI 1.22-1.69; ages 76 to 85 vs 45 to 65: OR 2.81; 95% CI 2.32-3.41 after adjusting for sex and race/ethnicity), whereas preference for obtaining information only from websites significantly declined with age (ages 66 to 75 vs 45 to 65: OR 0.77, 95% CI 0.66-0.89; ages 76 to 85 vs 45 to 65: OR 0.48; 95% CI 0.39-0.57). Similarly, among those who were interested in receiving health newsletters, preference for getting them only by mail significantly increased with age (ages 66 to 75 vs 45 to 65: OR 1.75, 95% CI 1.48-2.07; ages 76 to 85 vs 45 to 65: OR 4.05; 95% CI 3.31-4.95), whereas preference for getting them only by email declined with age (ages 66 to 75 vs 45 to 65: OR 0.60, 95% CI 0.51-0.70; ages 76 to 85 vs 45-65: OR 0.27; 95% CI 0.22-0.33). Compared with college graduates, after adjusting for age, sex, and race/ethnicity, adults with no college education or some college were significantly more likely to want print materials only (OR 2.11, 95% CI 1.72 to 2.58; OR 1.44, 95% CI 1.21-1.71, respectively) and mailed newsletters only (OR 2.83, 95% CI 2.30-3.48; OR 1.84, 95% CI 1.53-2.22, respectively), and significantly less likely to want emailed newsletters only (OR 0.41; 95% CI 0.33-0.51; OR 0.61, 95% CI 0.51-0.72, respectively); preference for internet-based materials only was not significantly associated with education.

**Table 4:** Preferred methods of obtaining health information and advice, by age group.

HIA^a,b^ modality			45 to 65 years (n=3671)^c^				66 to 75 years (n=2196)				76 to 85 years (n=1707)		
			%^c^		95% CI		%		95% CI		%		95% CI
**Any internet-based HIA modality^d^**			79.3		77.9-80.8		72.5^e^		70.4-74.6		55.2^e,f^		52.1-58.4
	Internet users^g^	81.3		79.9-82.8		78.6		76.6-80.6		69.8^e,f^		66.5-73.0	
**HIA from a website**			50.5		48.7-2.2		49.2		46.9-1.6		35.1^e,f^		32.1-38.1
	Internet users^g^	52.0		50.2-53.9		53.6		51.2-56.1		46.3^e,f^		42.7-49.9	
Web-based video^h^			24.2		22.7-25.8		15.3^e^		13.6-17.0		7.1^e,f^		5.7-8.5
Web-based interactive program^h^			12.1		11.0-13.3		7.3^e^		6.0-8.5		2.9^e,f^		2.0-3.8
Video visit with a patient educator^h^			8.0		7.0−8.9		4.6^e^		3.6−5.5		2.7^e,i^		1.7-3.7
**Message sent through the patient portal**			38.1		36.3-39.8		38.5		36.3-40.8		23.9^e,f^		21.2-26.5
	Internet users^g^	39.4		37.6-41.2		42.3		39.8-44.7		31.7^e,f^		28.4-35.0	
**Emailed newsletter**			37.3		35.5-39.0		38.7		36.4-41.0		27.2^e,f^		24.4-30.1
	Email users	38.6		36.8-40.4		42.7^j^		40.2-45.2		34.5^f,j^		31.0-37.9	
Podcast/audio download^h^			7.3		6.4-8.2		4.4^e^		3.4-5.4		2.6^e,i^		1.6-3.7
Webinar or Web-based talk^h^			9.6		8.6-10.7		6.1^e^		5.0-7.2		2.8^e,f^		1.7-3.8
Chat room/online health community^h^			3.4		2.7-4.0		1.1^e^		0.7-1.5		0.9^e^		0.4-1.5
**Health app**			22.5		21.0-24.0		10.9^e^		9.4-12.4		4.3^e,f^		3.2-5.5
	If has a smartphone	29.5		27.5-31.5		20.5^e^		17.6-23.3		14.9^e^		10.1-19.6	
**Any noninternet HIA modality^k^**			71.0		69.3-72.6		76.6^e^		74.6-78.6		82.4^e,f^		80.0-84.7
	Internet users^g^	70.1		68.4-71.8		74.7^e^		72.6-76.9		78.3^e,i^		75.5-81.1	
Print materials			33.1		31.4-34.7		39.1^e^		36.8-41.3		42.2^e^		39.1-45.4
In-person workshop or multi-session class			25.8		24.2-27.3		25.3		23.3-27.3		21.2^i,l^		18.7-23.8
Counseling/coaching over the phone			16.3		15.0-17.6		17.2		15.5-19.0		18.2		15.7-20.7
In-person individual counseling			32.1		30.4-33.7		29.9		27.8-32.0		29.4		26.6-32.3
Mailed newsletter			24.2		22.7-25.7		35.9^e^		33.6-38.1		48.4^e,f^		45.3-51.6
**Text message^m^**			18.7		16.7-20.6		15.7^j^		13.2-18.2		8.9^e,f^		6.7-11.1
	If has a mobile phone	19.1		17.1-21.1		16.5		13.9-19.1		10.6^e,n^		7.9-13.2	

^a^HIA: health information or advice.

^b^Prevalence of interest in using an HIA modality is estimated from weighted data for the 86% of the sample that indicated interest in using any HIA modality in the survey checklist.

^c^%: percentage of age group with this characteristic based on weighted survey data.

^d^Internet-based HIA: information from a webpage, Web-based video, Web-based interactive program, video visit, patient portal message, podcast, webinar/Web-based talk, or online community or chat room.

^e^Significantly (*P<*.001) lower than ages 45 to 65 years after controlling for sex and race/ethnicity.

^f^Significantly (*P<*.001) lower than ages 66 to 75 years after controlling for sex and race/ethnicity.

^g^Internet users are those who use the internet on their own or with help. Ns for internet users: ages 45 to 65 years: 1259; ages 66 to 75 years: 699; ages 76 to 85 years: 429.

^h^Prevalence of interest among internet users is not reported but differs from prevalence for all adults in the 76 to 85 year age group by less than 5% and by less than 2% points for all adults in the 2 younger age groups.

^i^Significantly (*P<*.05) lower than ages 66 to 75 years after controlling for sex and race/ethnicity.

^j^Significantly (*P<*.05) lower than ages 45 to 65 years after controlling for sex and race/ethnicity.

^k^Noninternet HIA: information from print materials, workshop/class, in-person or phone coaching, text message, or mailed newsletter.

^l^Significantly (*P<*.01) lower than ages 45 to 65 years after controlling for sex and race/ethnicity.

^m^Only asked about in the 2015 survey questionnaire. Subgroup Ns are approximately half as large as above.

^n^Significantly (*P<*.01) lower than ages 66 to 75 years after controlling for sex and race/ethnicity.

## Discussion

### Principal Findings

Although we have previously reported on disparities in use of digital technologies and interest in using internet-based health information resources by seniors in this health plan population [23], in this study, which used data from a more recent survey, we extend the comparison to middle-aged adults and focus on adults who are managing chronic health conditions. In this study, we showed that there are significant age-group and educational disparities in access to or use of digital technologies used to access internet-based HIA, including computers, smartphones, email, text messaging, and apps. Specifically, younger (aged 66 to 75 years) and older (aged 76 to 85 years) seniors were significantly less likely than middle-aged adults (aged 45 to 65 years) to be using these digital technologies, and older seniors were significantly less likely than younger seniors to be doing so. Within each age group, we showed that use of these digital technologies was significantly lower among adults who have no formal education beyond high school or some college as compared with college graduates. Similar disparities by age group, and education within age group, in access to and use of digital technologies were also observed in the 2017 CPS-CIUS [6].

Across all age groups, only about half of adults had sought health information from Web-based sources during the past year or were interested in doing so in the future. Although younger seniors were less likely than middle-aged adults to use the internet alone or with help, we did not observe similar age group differences with regard to having used the internet to obtain health information in the past year or interest in using an internet-based modality in the future. Older seniors were less likely than the younger 2 groups to be using the internet and also less likely to have used or be interested in using internet-based health information resources. When we restricted our analyses to internet users, the difference between the older senior group and younger 2 groups in seeking health information from the internet in the past year substantially diminished, but the older group remained less interested in using an internet-based health information resource in the future. We found significant disparities by education in past year use and interest in future use of internet-based health information resources, although within age groups, the differences between college graduates and those with some college were much smaller than differences between college graduates and those with no college education, and the latter differences were still smaller among internet users.

With regard to interest in using specific health information and health education modalities, we found that interest in using Web-based health information resources (webpage information, Web-based videos, interactive patient education programs, webinars, podcasts/online audio programs, online chat rooms/communities, emailed newsletters, messages sent through the patient portal, text messages, and video visits with a patient educator) was substantially lower among older seniors than among the middle-aged and younger senior groups. For example, among those who were interested in textual health information and health newsletters, some of the differences (eg, interest in information from a website, emailed newsletters, and patient portal messages) were associated with not being an internet user or email user. This was not the case for most of the online modalities, where there was very little difference in percentages of all adults and online adults who were interested in using the modalities. Within all 3 age groups, interest in the online modalities was significantly lower among those with no college education than among college graduates.

Across all age groups, the percentages of adults who expressed interest in using health apps and podcasts in the future were about twice as high as the percentages of adults who had reported using these modalities in the previous year. Prevalence of interest in using health apps was also twice as high between younger and older seniors who owned a smartphone compared with all adults in those age groups, suggesting that as smartphone ownership increases in these older age groups, there is potential for greater uptake of health apps. Interest in listening to health podcasts was very low and was not substantially higher among smartphone owners than all adults for any age group.

The percentages of middle-aged, younger senior, and older senior adults in our study population who in 2014 used the internet to obtain health information were comparable with those observed in national samples for middle-aged and older adults [58] but substantially higher than estimates for all US adults in these age groups on the basis of the 2015 CPS-CIUS population (51.9% vs 39.2%, 51.1% vs 31.5%, and 38.1% vs 23.3%, respectively) [5]. This difference in internet-based health information seeking can be partially explained by differences in population demographics. Across all 3 age groups, compared with the US population, this study’s population had higher percentages of adults who had attended some college or were college graduates and lower percentages with lower household incomes (>US $35,000) [5]. As numerous studies have shown that use of the internet increases as educational attainment and HHI increase, it is not surprising that this study’s population had a higher proportion of internet users in all 3 age groups than the US population (approximately 95% vs 76%, 88% vs 64%, and 68% vs 42%, respectively) and thus had greater capability to search for health information online. When we restricted our comparison of these age groups to internet users, we found that the percentages in this study’s population who had sought health information from the internet were only slightly higher than among these same age groups in the US population (52.4% vs 51.1%, 55.5% vs 50.2%, and 53.0% vs 45.1%, respectively) [5].

Another potential reason for the higher prevalence of internet-based health information seeking in our health plan population is that this study’s population was restricted to adults who had at least one chronic health condition, and previous research has found that adults with chronic conditions are more likely to use patient portals and Web-based patient education resources [8,9,32,33,59,60]. The percentages of middle-aged and older adults who used the internet to obtain health information in the past year estimated from our survey and the 2015 CPS supplement are much lower than those reported in a 2012 Pew survey (71% of middle-aged and 58% of adults aged ≥65 years, who used the internet and 54% and 30%, respectively, of all adults in those age groups) [3].

The results of this study and other research suggest that when planning delivery of health information and patient education for adults with chronic health conditions, it is important to take into account the population’s age group composition and educational attainment to gauge the likely uptake of internet-based and mobile health (mHealth; mobile technology-based) resources. Although more middle-aged and older adults are using the internet now than in the past [61], they are still less likely than younger adults to be using the internet and using the internet for functions other than email [7,9,14]. Many noninternet users lack easy access to digital technology (internet-enabled devices, high speed internet connections) that could connect them to the internet [8,12], and many older adults with chronic health conditions have physical or cognitive impairments that make it difficult to use internet-based resources [11,16,62]. In addition, even those currently using the internet might lack internet-based health skills (ability to access and use DITs for health purposes), experience, comfort, and trust in accessing internet-based health information resources [4,8,63]. For various reasons, they might also just prefer to get health information through print materials or directly from a person rather than from an internet-based source [23,32,39-41]. As adults with lower levels of educational attainment are less likely to seek health information in general, let alone use the internet to do so [64], it is important to make sure that HIA remains easily available through modalities that noninternet using adults will be more likely to use.

Without encouragement and support from health care professionals, family, and friends, middle-aged and older adults with chronic conditions who are not currently using internet-based health resources and health apps are unlikely to make the transition to electronic health and Web 2.0 [37]. However, even with encouragement, these adults are likely going to need assistance in gaining access to Web-enabled computers and other digital devices that they can comfortably use to connect with, navigate, and read information on the internet, as well as use high-speed internet or Wi-Fi if they have their own devices. Although younger adults find smartphones and tablets work well for performing online functions, aging adults with poorer vision and less manual dexterity might need to use a desktop or laptop computer with a larger screen and manual keyboard. They will also likely need training and support in how to use these digital tools, navigate the internet, conduct Web searches, and download materials [4]. Most public libraries offer access to computers and printers, Wi-Fi for people who bring their own Web-enabled devices, and librarians or volunteers to assist those who need help with online tasks [65]. Many libraries and community centers also offer classes for adults in how to use different types of digital devices and interact with the internet [66].

Developers of Web-based health information resources and health apps must also test their products with a wide range of potential end-users to make sure that these programs and tools are both effective in what they aim to achieve and easy for older and less educated adults to use [37]. In addition, health care providers and patient educators should not assume that even patients who are using a patient portal or are college-educated will follow up on recommendations to access Web-based health resources. Some patients, who might be willing to use Web-based and mHealth patient education and self-management tools but lack the equipment to do so, might also need financial assistance to purchase digital technology or to be given access to loaner equipment.

### Strengths

This study has a number of strengths. First, the survey dataset enabled us to estimate the prevalence of use of multiple DITs and interest in using several different internet-based and mHealth modalities to obtain HIA in a population of insured patients with chronic health conditions. Second, because of the large sample size and sociodemographic diversity of the study cohort, we were able to show significant disparities in use of DITs and health information modality preferences across 3 age groups (middle-aged, younger seniors, and older seniors) and by education within age groups using directly observed weighted percentages, not just ORs from logistic regression models. Third, we were able to show how prevalence of previous use of and future interest in using different types of internet-based health information resources differed by age group and education among the segment of this patient population that was using the internet.

### Limitations

The survey was conducted with adults from 1 Northern California health plan membership that, while fairly representative of Northern California adults, is not representative of the US middle-aged and older adult population with regard to educational attainment, income, broadband internet access, and health care coverage. The health plan membership is better educated and has a lesser percentage of low-income adults than the general US adult population and primarily resides in urban and suburban communities with widespread access to home and workplace broadband internet and free Wi-Fi in commercial and community settings. Moreover, members of this health plan are encouraged by the health care staff to use the comprehensive health information and health education resources available on the health plan’s website. The confluence of these sociodemographic and internet-related factors might have increased the percentages of adults in all 3 age groups who used DITs and are interested in going online for health information. The survey did not include adults with a primary language other than English and with no health care coverage, and it did not include groups whose ability to access and preferences for using internet-based health information resources might differ from thus study’s population and thus limit generalizability to the entire US population. This study’s sample excluded adults who were missing data on internet use status. However, the percentages of respondents with missing data for this variable were so small (0.8%, 1.8%, and 3.7% of middle-aged, younger senior, and older senior adults, respectively, after weighting) that we do not believe this introduced much bias in the results. Finally, although we used logistic regression models to control for race/ethnicity and sex when we tested for age group and educational disparities in DIT use and health information modality preferences, we did not examine whether the same patterns of disparity were found across all race/ethnic groups. An earlier survey of seniors in this health plan membership found that within level of education, black and Latino seniors were less likely than non-Hispanic white and Asian seniors to be using the health plan’s patient portal [8,23]. Future research is needed to identify whether sociodemographic and sociocultural factors differentially influence use of DITs and preferences for using specific internet-based and mHealth information modalities among adults in different racial/ethnic groups. Such information would improve the evidence base for development and implementation of patient-centered resources at the population level to prevent chronic health conditions and improve CCM, health outcomes, and quality of life.

### Conclusions

DITs and internet-based health information resources provide a relatively inexpensive and effective way for adults with chronic health conditions to access information that can help them learn about and manage their health. However, this study found significant digital divides by age and educational attainment among middle-aged and older adults in ownership of digital devices and preferences for using internet-based resources to obtain HIA. These digital divides could potentially limit access to valuable health information and chronic disease self-management resources among vulnerable adult populations. Bridging digital divides in use of internet-based health resources will require ongoing personal encouragement from clinical staff for patients to try these new resources, including talking up the advantages of using these as an adjunct to and not replacement of aspects of the way they currently receive health care and obtain and share health information that they value. Patients reluctant to engage with digital information resources might also need to be provided with print materials and personal (nonvirtual) learning opportunities to become comfortable using these resources. Health care providers and consumer health organizations should also user test their internet-based resources before implementation to make sure that older and less educated adults will be able to use them easily and effectively. Finally, as part of providing patient-centered care, it will be important for health care providers and other consumer health organizations to continue to make it easy for patients to obtain health information and patient education from print materials, one-on-one patient counseling, and other more traditional modalities.

## Appendix

Multimedia Appendix 1 Logistic regression models predicting use of Web-based health information and advice resources in past 12 months, aged 45 to 85 years and age groups 45 to 65 years, 66 to 75 years, and 75 to 85 years.

Multimedia Appendix 2 Preferred methods of obtaining health information and advice by level of education, adults aged 45 to 65, 66 to 75, and 76 to 85 years.

## Abbreviations

CCM: chronic conditions management
CPS-CIUS: US Current Population Survey Computer and Internet Use Supplement
DIT: digital information technology
DVD: digital versatile disc
HHI: household income
HIA: health information and advice
KPNC: Kaiser Permanente Medical Care Program in Northern California
mHealth: mobile health
MHS: Member Health Survey
OR: odds ratio
