# Supplementary material for: Digital Information Technology Use and Patient Preferences for Internet-Based Health Education Modalities: Cross-Sectional Survey Study of Middle-Aged and Older Adults With Chronic Health Conditions
Source: JMIR Aging. 2019 Apr 4;2(1):e12243. doi: 10.2196/12243 (PMC6716442; doi:10.2196/12243)
Supplement: Multimedia Appendix 1 [file aging_v2i1e12243_app1.pdf]

MULTIMEDIA APPENDIX 1:

Logistic regression models predicting use of Web-based HIA[a] resources in past 12 months, ages 45 to 85 and age groups 45 to 65, 66 to 75, and 75 to 85 years

	All		Internet Users	
	AOR (95% CI)[a]	*P-value*	AOR (95% CI)	*P-value*
**Ages 45 to 85 years**				
Sex				
Male	(ref)		(ref)	
Female	1.24 (1.12-1.37)	<.001	1.27 (1.14-1.41)	<.001
Age group				
45-65 yr	(ref)		(ref)	
66-75 yr	0.91 (0.81-1.01)	0.08	1.07 (0.95-1.20)	0.29
76-85 yr	0.59 (0.51-0.67)	<.001	0.99 (0.85-1.16)	0.90
Race/ethnicity				
White non-Hispanic	(ref)		(ref)	
Black	0.76 (0.63-0.92)	0.004	0.86 (0.70-1.06)	0.16
Hispanic	0.64 (0.54-0.76)	<.001	0.68 (0.57-0.82)	<.001
Filipino	0.56 (0.36-0.88)	0.011	0.62 (0.39-0.98)	0.040
East Asian	0.64 (0.42-0.98)	0.041	0.64 (0.41-0.99)	0.045
Other Asian	0.94 (0.63-1.38)	0.73	0.98 (0.65-1.46)	0.91
Other race/ethnicity	0.88 (0.62-1.24)	0.46	0.94 (0.66-1.36)	0.77
Education				
College graduate	(ref)		(ref)	
Some college	0.82 (0.73-0.92)	0.001	0.90 (0.80-1.02)	0.11
No college	0.48 (0.42-0.55)	<.001	0.66 (0.57-0.77)	<.001
**Ages 45 to 65 years**				
Sex				
Male	(ref)		(ref)	
Female	1.43 (1.24-1.64)	<.001	1.42 (1.23-1.63)	<.001
Race/ethnicity				
White non-Hispanic	(ref)		(ref)	
Black	0.79 (0.62-1.00)	0.05	0.87 (0.68-1.12)	0.29
Hispanic	0.64 (0.52-0.79)	<.001	0.66 (0.54-0.82)	<0.001
Filipino	0.56 (0.32-0.97)	0.037	0.57 (0.32-1.00)	0.05
East Asian	0.54 (0.32-0.91)	0.022	0.51 (0.30-0.88)	0.016
Other Asian	1.05 (0.65-1.70)	0.83	1.11 (0.68-1.81)	0.68
Other race/ethnicity	0.91 (0.58-1.43)	0.69	0.97 (0.61-1.55)	0.90
Education				
College graduate	(ref)		(ref)	
Some college	0.87 (0.75-1.02)	0.09	0.93 (0.80-1.10)	0.40
No college	0.57 (0.47-0.68)	<.001	0.69 (0.57-0.83)	<0.001

(contd.)

Logistic regression models predicting use of Web-based HIA[a] resources in past 12 months, ages 45 to 85 and age groups 45 to 65, 66 to 75, and 75 to 85 years (contd.)

	All		Internet Users	
	AOR (95% CI)[a]	*P-value*	AOR (95% CI)	*P-value*
**Ages 66 to 75 years**				
Sex				
Male	(ref)		(ref)	
Female	1.05 (0.88-1.25)	0.61	1.05 (0.87-1.26)	0.63
Race/ethnicity				
White non-Hispanic	(ref)		(ref)	
Black	0.71 (0.48-1.03)	0.61	0.89 (0.59-1.34)	0.576
Hispanic	0.74 (0.53-1.03)	0.07	0.83 (0.57-1.20)	0.32
Filipino	0.52 (0.24-1.12)	0.07	0.73 (0.33-1.62)	0.44
East Asian	1.15 (0.53-2.48)	0.09	1.34 (0.61-2.93)	0.46
Other Asian	0.74 (0.37-1.47)	0.73	0.71 (0.36-1.40)	0.327
Other race/ethnicity	0.71 (0.41-1.23)		0.81 (0.44-1.48)	0.50
Education				
College graduate	(ref)	0.39	(ref)	
Some college	0.78 (0.64-0.95)	0.22	0.91 (0.74-1.12)	0.37
No college	0.35 0.27-0.44)	<.001	0.52 (0.40-0.68)	<.001
**Ages 76 to 85 years**				
Sex				
Male	(ref)		(ref)	
Female	0.80 (0.62-1.01)	0.062	0.89 (0.68-1.16)	0.39
Race/ethnicity				
White non-Hispanic	(ref)		(ref)	
Black	0.63 (0.36-1.07)	0.09	0.67 (0.37-1.20)	0.17
Hispanic	0.43 (0.29-0.65)	<.001	0.57 (0.36-0.89)	0.014
Filipino	0.82 (0.26-2.65)	0.74	0.97 (0.27-3.49)	0.96
East Asian	1.05 (0.34-3.26)	0.94	1.22 (0.36-4.15)	0.75
Other Asian	0.52 (0.19-1.46)	0.21	0.53 (0.18-1.60)	0.26
Other race/ethnicity	0.97 (0.44-2.15)	0.94	1.04 (0.41-2.60)	0.94
Education				
College graduate	(ref)		(ref)	
Some college	0.60 (0.45-0.80)	0.015	0.72 (0.52-0.98)	0.038
No college	0.36 (0.26-0.48)	<.001	0.86 (0.61-1.21)	0.38

[a]HIA: health information and advice; AOR: adjusted odds ratio; CI: confidence interval
