# Supplementary material for: Digital Information Technology Use and Patient Preferences for Internet-Based Health Education Modalities: Cross-Sectional Survey Study of Middle-Aged and Older Adults With Chronic Health Conditions
Source: JMIR Aging. 2019 Apr 4;2(1):e12243. doi: 10.2196/12243 (PMC6716442; doi:10.2196/12243)
Supplement: Multimedia Appendix 2 [file aging_v2i1e12243_app2.pdf]

MULTIMEDIA APPENDIX 2:

Table 5. Preferred methods of obtaining health information and advice, adults aged 45 to 65 years, by level of education[a]

HIA modality	College graduate (N=1,574)		Some college (N=1,311)		No college (N=772)	
	%	95% CI	%	95% CI	%	95% CI
Any internet-based HIA modality	85.4	83.4-87.4	78.0[b]	75.6-80.4	69.2[b,c]	65.6-72.7
Internet users[d]	85.7	83.7-87.7	79.9[b]	77.4-82.3	74.3[b,e]	70.6-77.9
HIA from a webpage	55.5	52.7-58.2	50.2[f]	47.2-53.1	40.7[b,c]	36.8-44.6
Internet users[d]	55.7	53.0-58.5	51.8	48.8-54.9	44.2[b,g]	40.1-48.4
Web-based video	28.7	26.2-31.2	22.9[b]	20.3-25.4	17.1[b,i]	14.1-20.0
Web-based interactive program[h]	15.9	13.9-18.0	11.1[b]	9.2-12.9	5.9[b,j]	4.0-7.8
Video visit with a patient educator[h]	10.8	9.2-12.5	6.9[b]	5.4-8.5	3.6[b,l]	2.1-5.0
Message sent through the patient portal	40.9	38.2-43.6	38.6	35.7-41.5	31.5[b,g]	27.8-35.2
Internet users[d]	41.1	38.4-43.9	39.9	36.9-42.8	34.6[f]	30.6-38.6
Emailed newsletter	42.0	39.3-44.8	35.4[o]	32.5-38.2	30.9[b]	27.3-34.6
Email users	42.4	39.6-45.1	36.6[e]	33.6-39.5	34.2[p]	30.1-38.2
Podcast/audio download[h]	10.4	8.7-12.0	5.9[b]	4.6-7.3	3.3[b]	2.0-4.7
Webinar or Web-based talk[h]	12.4	10.5-14.2	9.3[q]	7.6-11.0	4.5[b,c]	2.8-6.1
Chat room/online health community[h]	3.5	2.5-4.5	3.5	2.4-4.5	2.7	1.3-4.1
Health app	26.5	24.1-29.0	22.4	19.9-24.9	14.2[b,c]	11.5-17.0
If has a smartphone	32.6	29.6-35.6	29.2	25.9-32.5	21.4[b,r]	17.1-25.7
Any non-internet HIA modality	69.2	66.6-71.8	72.1	69.5-74.8	72.4	68.9-75.9
Print materials	32.0	29.4-34.6	35.7	32.9-38.6	31.0	27.4-34.6
In-person workshop or multi-session class	29.4	26.9-31.9	24.5[b]	22.0-27.0	20.5[b]	17.2-23.8
Counseling/coaching over the phone	17.3	15.2-19.3	15.5	13.4-17.7	15.5	12.7-18.3
In-person individual counseling	33.8	31.2-36.5	32.1	29.3-34.9	28.1[b,k]	24.6- 31.6
Mailed newsletter	18.9	16.9-21.0	27.0[b]	24.4-29.6	31.1[b]	27.5-34.7
Text message[m]	16.6	13.7-19.4	21.4	17.9-24.9	18.9	14.7-23.1
Mobile phone users	17.0	14.0-19.9	21.9	18.3-25.4	19.3	15.0-23.5

N: unweighted count; %: percentage of age group with this characteristic based on weighted survey data; CI: confidence interval; HIA: health information or advice; internet-based HIA modalities: information from a webpage, Web-based videos, Web-based interactive programs, video visit, patient portal messages, podcasts, webinars/Web-based talks, and chat rooms; non-internet HIA modalities: print materials, classes/workshops, in-person or phone counseling, mailed newsletters, and text messages.

[a] Prevalence of interest in using an HIA modality is estimated from weighted data for the 86% of the sample that indicated interest in using any HIA modality in the survey checklist.

[b] Significantly (*P* < .001) lower than college graduates after controlling for sex and race/ethnicity.

[c] Significantly (*P* < .001) lower than adults with some college after controlling for sex and race/ethnicity.

[d] Internet users are those who use the internet on their own or with help. Ns for internet users: no college: 683; some college: 1,259; college graduates: 1,563.

[e] Significantly (*P* < .02) lower than adults with some college after controlling for sex and race/ethnicity.

[f] Significantly (*P* < .001) lower than college graduates after controlling for sex and race/ethnicity.

[g] Significantly (*P* < .001) lower than adults with some college after controlling for sex and race/ethnicity.

[h] Prevalence of interest among internet users is not reported but differs from prevalence for all adults in this age group by < 2 percentage points.

[i] Significantly (*P* = .002) lower than adults with some college after controlling for sex and race/ethnicity.

[j] Significantly (*P* = .016) lower than adults with some college after controlling for sex and race/ethnicity.

[k] Significantly (*P* < .04) lower than adults with some college after controlling for sex and race/ethnicity.

[l] Significantly (*P* = .003) lower than adults with some college after controlling for sex and race/ethnicity.

[m] Only asked about in the 2015 survey questionnaire. Subgroup Ns approximately half as large as above.

[n] Significantly (*P* = .011) lower than college graduates after controlling for sex and race/ethnicity.

[o] Significantly (*P* = .003) lower than college graduates after controlling for sex and race/ethnicity.

[p] Significantly (*P* = .004) lower than college graduates after controlling for sex and race/ethnicity.

[q] Significantly (*P* = .006) lower than college graduates after controlling for sex and race/ethnicity.

[r] Significantly (*P* = .007) lower than adults with some college after controlling for sex and race/ethnicity.

MULTIMEDIA APPENDIX 2 (contd).

Table 6. Preferred methods of obtaining health information and advice, adults aged 66 to 75 years, by level of education[a]

HIA modality	College graduate (N=928)		Some college (N= 778)		No college (N=475)	
	%	95% CI	%	95% CI	%	95% CI
Any internet-based HIA modality	81.2	78.5-83.9	71.3 [b]	67.7-74.9	54.7 [b,c]	49.6-59.8
Internet users[d]	83.2	80.6-85.9	77.6 [b]	74.1-81.1	67.3 [b,c]	61.8-72.8
HIA from a webpage	57.9	54.4-61.4	47.9 [b]	43.9-51.8	31.4 [b,c]	26.7-36.2
Internet users[d]	59.5	56.0-63.1	52.0 [e]	47.8-56.1	39.8 [b,f]	34.1-45.5
Web-based video [g]	19.3	16.4-22.1	13.3 [h]	10.6-16.0	10.2 [b,c]	7.1-13.3
Web-based interactive program[g]	10.1	7.9-12.3	6.3 [i]	4.4-8.3	2.7 [b,c]	0.9-4.4
Video visit with a patient educator[g]	6.6	4.8-8.3	3.9 [j]	2.4-5.4	1.3 [b,f]	0.1-2.5
Message sent through the patient portal	43.6	40.1-47.2	38.8	35.0-42.7	26.4 [b,c]	21.8-31.0
Internet users[d]	45.0	41.4-48.6	42.6	38.5-46.7	33.5 [l,f]	27.8-39.1
Emailed newsletter	46.0	42.4- 49.5	36.1 [b]	32.3-39.9	27.1 [b,c]	22.5-31.7
Email users	47.2	43.5-50.8	40.3 [l]	36.2-44.5	34.3 [b]	28.5-40.1
Podcast/audio download[g]	5.8	4.1-7.6	4.3	2.7-5.9	1.6 [n]	0.3-2.9
Webinar or Web-based talk[g]	8.4	6.5-10.4	5.0 [e]	3.2-6.9	2.9 [b]	1.2-4.5
Chat room/online health community[g]	1.4	0.7-2.2	0.9	0.2-1.5	0.8	<0.1-1.7
Health app	13.3	10.9-15.7	11.2	8.7-13.8	4.6 [b,c]	2.6-6.7
If has a smartphone	20.8	16.9-24.7	22.1	17.0-27.1	12.5 [o,p]	6.1-19.0
Any non-internet HIA modality	76.4	73.5-79.4	75.6	72.2-79.0	78.5	74.3-82.8
Print materials	41.0	37.5-44.5	39.0	35.1-42.8	34.8	29.9-39.6
In-person workshop or multi-session class	31.3	28.1-34.6	24.4 [b,c]	20.9-27.8	13.5 [b,c]	10.2-16.9
Counseling/coaching over the phone	17.0	14.3-19.6	15.8	12.9-18.6	20.3	16.2-24.3
In-person individual counseling	32.7	29.4-36.0	29.2	25.6-32.7	25.9 [j]	21.4-30.4
Mailed newsletter	32.4	29.1-35.7	35.1	31.3-38.8	44.4 [b,m]	39.4-49.4
Text message[k]	17.7	13.7-21.7	13.3	9.4-17.1	13.8	8.8-18.8
Mobile phone users	18.2	14.0-22.4	14.1	10.0-18.2	15.0	9.7-20.4

N: unweighted count; %: percentage of age group with this characteristic based on weighted survey data; CI: confidence interval; HIA: health information or advice; internet-based HIA modalities: information from a webpage, Web-based videos, Web-based interactive programs, video visit, patient portal messages, podcasts, webinars/Web-based talks, and chat rooms; non-internet HIA modalities: print materials, classes/workshops, in-person or phone counseling, mailed newsletters, and text messages.

[a]Prevalence of interest in using an HIA modality is estimated from weighted data for the 86% of the sample that indicated interest in using any HIA modality in the survey checklist.

[b]Significantly (P < .001) lower than college graduates after controlling for sex and race/ethnicity.

[c] Significantly (P < .001) lower than adults with some college after controlling for sex and race/ethnicity.

[d] Internet users are those who use the internet alone or with help. Ns for internet users: no college: 346; some college:699; college graduate: 895.

[e] Significantly (P < .007) lower than adults with some college after controlling for sex and race/ethnicity.

[f] Significantly (P < .03) lower than adults with some college after controlling for sex and race/ethnicity.

[g] Prevalence of interest among internet users is not reported but differs from prevalence for all adults in this age group by < 2 percentage points.

[h] Significantly (P = .001) lower than college graduates after controlling for sex and race/ethnicity.

[i] Significantly (P = .002) lower than college graduates after controlling for sex and race/ethnicity.

[j] Significantly (P = .01) lower than college graduates after controlling for sex and race/ethnicity.

[k] Only asked about in the 2015 survey questionnaire. Subgroup Ns are approximately half as large as above.

[l] Significantly (P < .02) lower than college graduates after controlling for sex and race/ethnicity.

[m] Significantly (P = .009) lower than adults with some college after controlling for sex and race/ethnicity.

[n] Significantly (P < .002) lower than college graduates after controlling for sex and race/ethnicity.

[o] Significantly (P < .001) lower than college graduates after controlling for sex and race/ethnicity.

[p] Significantly (P = .038) lower than adults with some college after controlling for sex and race/ethnicity.

MULTIMEDIA APPENDIX 2 (contd):

Table 7. Preferred methods of obtaining health information and advice, adults aged 76 to 85 years, by level of education[a]

HIA modality	College graduate (N=620)		Some college (N=533)		No college (N=540)	
	%	95% CI	%	95% CI	%	95% CI
Any internet-based HIA modality	65.1	60.0-70.2	59.5	54.0-65.0	42.4[b,c]	36.7-48.1
Internet users[d]	72.1	67.1-77.2	71.5	66.0-77.0	64.6	57.8-71.5
HIA from a webpage	42.9	37.7-48.1	37.1	31.6-42.5	25.8[b,c]	20.9-30.7
Internet users[d]	47.7	42.2-53.2	45.4	39.2-51.6	45.7	38.4-53.1
Web-based video[g]	11.4	8.5-14.3	6.9[e]	4.6-9.2	3.1[b,f]	1.3-4.8
Web-based interactive program[g]	5.8	3.6-8.1	2.3[h]	0.7-3.9	0.5[b,i]	<0.1-1.0
Video visit with a patient educator[g]	3.7	1.9-5.5	2.2	0.7-3.6	2.1	0.2-3.9
Message sent through the patient portal	30.8	26.1-35.4	24.6	19.9-29.4	16.5[b,l]	12.0-21.0
Internet users	34.1	29.1-39.2	31.3	25.6-37.1	28.3	21.5-35.1
Emailed newsletter	33.7	28.7-38.6	28.0	23.1-33.0	20.6[o]	15.8-25.3
Email users	38.2	32.7-43.6	34.0	28.0-40.1	29.0	22.3-35.8
Podcast/audio download[g]	4.9	2.3-7.4	1.5[p]	0.4-2.6	1.3[b]	0.2-2.5
Webinar or Web-based talk[g]	4.9	2.5-7.2	2.3	0.7-4.0	1.0[e]	<0.1-2.4
Chat room/online health community[g]	1.8	0.3-3.2	0.6	0.0-1.2	0.4	<0.1-1.1
Health app	5.6	3.6-7.5	4.2	2.1-6.3	3.1	1.2-5.0
If has a smartphone	14.6	8.7-20.5	12.5	4.2-20.8	18.1	4.5-31.7
Any non-internet HIA modality	82.4	78.8-86.0	80.4	76.1-84.7	84.0	79.6-88.5
Print materials	46.4	41.2-51.7	40.7	35.1-46.2	39.4[e]	33.9-45.0
In-person workshop or multi-session class	29.3	24.4-34.1	20.5[j]	16.3-24.6	14.0[b,f]	10.0-17.9
Counseling/coaching over the phone	21.0	16.5-25.4	14.0[k]	10.4-17.6	18.7	14.2-23.2
In-person individual counseling	35.1	30.1-40.1	26.7[h]	21.8-31.5	26.0[h]	21.0-31.0
Mailed newsletter	44.0	38.7-49.2	49.4	43.8-55.0	52.2	46.5-57.9
Text message[m]	13.3	8.8-17.8	7.4	3.8-10.9	5.7	2.6-8.8
Mobile phone users	14.8	9.8-19.8	8.6	4.4-12.7	7.5	3.4-11.6

N: unweighted count; %: percentage of age group with this characteristic based on weighted survey data; CI: confidence interval; HIA: health information or advice; internet-based HIA modalities: information from a webpage, Web-based videos, Web-based interactive programs, video visit, patient portal messages, podcasts, webinars/Web-based talks, and chat rooms; non-internet HIA modalities: print materials, classes/workshops, in-person or phone counseling, mailed newsletters, and text messages.

[a] Prevalence of interest in using an HIA modality is estimated from weighted data for the 86% of the sample that indicated interest in using any HIA modality in the survey checklist.

[b] Significantly (*P* < .001) lower than college graduates after controlling for sex and race/ethnicity.

[c] Significantly (*P* < .001) lower than adults with some college after controlling for sex and race/ethnicity.

[d] Internet users are those who use the internet alone or with help. Ns for internet users: no college: 314; some college: 429; college graduate: 557.

[e] Significantly (*P* < .04) lower than college graduates after controlling for sex and race/ethnicity.

[f] Significantly (*P* < .03) lower than adults with some college after controlling for sex and race/ethnicity.

[g] Prevalence of interest among internet users is not reported but differs from prevalence for all adults in this age group by < 5 percentage points.

[h] Significantly (*P* < .03) lower than college graduates after controlling for sex and race/ethnicity.

[i] Significantly (*P* < .017) lower than adults with some college after controlling for sex and race/ethnicity.

[j] Significantly (*P* = .001) lower than college graduates after controlling for sex and race/ethnicity.

[k] Significantly (*P* = .0073) lower than college graduates after controlling for sex and race/ethnicity.

[l] Significantly (*P* = .04) lower than adults with some college after controlling for sex and race/ethnicity.

[m] Only asked about in the 2015 survey questionnaire. Subgroup Ns are approximately half as large as above.

[n] Significantly (*P* = .04) lower than college graduates after controlling for sex and race/ethnicity.

[o] Significantly (*P* = .002) lower than college graduates after controlling for sex and race/ethnicity.

[p] Significantly (*P* = .004) lower than college graduates after controlling for sex and race/ethnicity.
